# Novel Design on Knee Exoskeleton with Compliant Actuator for Post-Stroke Rehabilitation

**DOI:** 10.3390/s25010153

**Published:** 2024-12-30

**Authors:** Lin Wu, Chao Wang, Jiawei Liu, Benjian Zou, Samit Chakrabarty, Tianzhe Bao, Sheng Quan Xie

**Affiliations:** 1 Institute of Robotics, Autonomous System and Sensing, School of Electronic and Electrical Engineering, University of Leeds, Leeds LS2 9JT, UK; ellw@leeds.ac.uk; 2 Wuhan Institute of Healthcare Tech Industry, Wuhan 430205, China; 3 School of Mechatronic Engineering and Automation, Shanghai University, Shanghai 200444, China; elcw@shu.edu.cn; 4 School of Rehabilitation Science and Engineering, University of Health and Rehabilitation Sciences, Qingdao 266114, China; liu1575979@163.com (J.L.); sduzoubenjian@163.com (B.Z.); 5 School of Biomedical Sciences, University of Leeds, Leeds LS2 9JT, UK; s.chakrabarty@leeds.ac.uk

**Keywords:** knee exoskeleton, series elastic actuator, robot-assisted therapies, stroke rehabilitation, knee joint disorders

## Abstract

Knee joint disorders pose a significant and growing challenge to global healthcare systems. Recent advancements in robotics, sensing technologies, and artificial intelligence have driven the development of robot-assisted therapies, reducing the physical burden on therapists and improving rehabilitation outcomes. This study presents a novel knee exoskeleton designed for safe and adaptive rehabilitation, specifically targeting bed-bound stroke patients to enable early intervention. The exoskeleton comprises a leg splint, thigh splint, and an actuator, incorporating a series elastic actuator (SEA) to enhance torque density and provide intrinsic compliance. A variable impedance control method was also implemented to achieve accurate position tracking of the exoskeleton, and performance tests were conducted with and without human participants. A preliminary clinical study involving two stroke patients demonstrated the exoskeleton’s potential in reducing muscle spasticity, particularly at faster movement velocities. The key contributions of this study include the design of a compact SEA with improved torque density, the development of a knee exoskeleton equipped with a cascaded position controller, and a clinical test validating its effectiveness in alleviating spasticity in stroke patients. This study represents a significant step forward in the application of SEA for robot-assisted rehabilitation, offering a promising approach to the treatment of knee joint disorders.

## 1. Introduction

The increasing prevalence of knee joint disorders is now a significant global health concern, impacting both the elderly and those with various neurological or musculoskeletal conditions [1,2,3]. Although conventional rehabilitation techniques have proven effective, they are often hindered by the scarcity of skilled therapists required for consistent and repetitive manual training [4]. In response, robot-assisted rehabilitation systems, especially exoskeletons, are developed for delivering repetitive and physically intensive rehabilitation exercises with enhanced intensity and accuracy. These systems not only facilitate motor recovery, but also provide quantitative measurements of patient progress, thereby improving functional independence while also alleviating the physical demands on therapists [5,6].

As a typical human-in-the-loop process, ensuring the safety and comfort of patients during their interaction with knee exoskeletons is critical for both their immediate well-being and the long-term success of robot-assisted training. A significant focus has been on designing highly backdriveable and compliant robots to enhance physical human–robot interaction (pHRI), even in scenarios involving power losses [7,8,9,10]. Brahmia et al. developed a knee reeducator for open muscular chain exercises, featuring a motorised linear actuator and a kinematic structure for controlled knee flexion and extension [11]. In this context, a critical aspect of exoskeleton design is to avoid misalignments that can occur due to the complex kinematics of human joints and variations in limb placement across therapy sessions [9,10]. To address this challenge, recent studies have introduced knee exoskeletons that incorporate advanced mechanical design and control strategies. For example, Beyl et al. introduced KNEXO, a powered knee exoskeleton that uses lightweight and compliant pleated pneumatic artificial muscles with a proxy-based sliding mode control. This design demonstrated low actuator torques during unassisted walking and provided effective, safe guidance in the assisted mode [12]. Moreover, advancements in linkage mechanisms, such as four-bar and five-bar linkages [13], have been well studied in enhancing the ergonomic alignment and functionality of knee exoskeletons [14,15,16,17].

Currently, robot compliance is increasingly recognised as a fundamental approach in enhancing pHRI systems [18,19]. Previous studies have proposed many types of actuators to achieve compliant interaction between robots and the environment, with series elastic actuators (SEAs) gaining increasing attention due to their unique capacity to offer compliance and precise force control capabilities [20]. Despite the aforementioned efforts, existing torque control methodologies for SEAs only consider the motor-side dynamics and load-side position but overlook the intricate and unknown dynamics on the load side [21,22]. For instance, Losey et al. define the desired closed-loop torque performance using model reference adaptive control (MRAC) without knowing the motor-side parameters. Many studies utilise an observer to estimate the disturbance torque, i.e., disturbance observer (DOB), which, however, may lead to unstable torque tracking errors due to the time-varying load-side dynamics [23,24,25]. Lin et al. presented an adaptive robust controller (ARC) for the torque control of an SEA based on modelling both load-side and motor-side dynamics [26]. However, the SEAs with a non-linear stiffness profile are not modelled. These exoskeletons are developed for the patients’ gait training, which requires them to have a basic walking ability. However, many early-stage stroke patients cannot move themselves voluntarily at all but still need physical training intervention.

Thereby, this study endeavours to design an SEA with inherent compliance to ensure the intrinsic safety of a knee exoskeleton for early-stage stroke patients. A variable impedance control method was then developed to enable the safe and adaptive rehabilitation therapy. A preliminary clinical study was conducted to evaluate the effectiveness of this knee exoskeleton, especially in reducing muscle spasticity in stroke patients. Two male participants with lower limb spasticity underwent passive knee flexion using the exoskeleton at slow and fast velocities. The sEMG signals from key knee extensor muscles were recorded and analysed to assess muscle tone changes. Experimental results showed a significant reduction in muscle tone over time, particularly at the faster velocity, indicating the exoskeleton’s potential in alleviating spasticity. The contributions of this article are summarised as two-fold: (1) development of a novel knee exoskeleton composed of an SEA; (2) a clinical test to verify the applicability of a knee exoskeleton in reducing the spasticity of stroke patients.

This paper is organised as follows: Section 2 introduces the design and control system of the proposed exoskeleton. Experimental results for the exoskeleton and clinical test are presented in Section 3, which are discussed in Section 4. Section 5 summarises the limitations of this study. Finally, Section 6 concludes this paper.

## 2. Methodology

### 2.1. Exoskeleton Design

#### 2.1.1. Structure of Exoskeleton Platform

**Figure 1:**
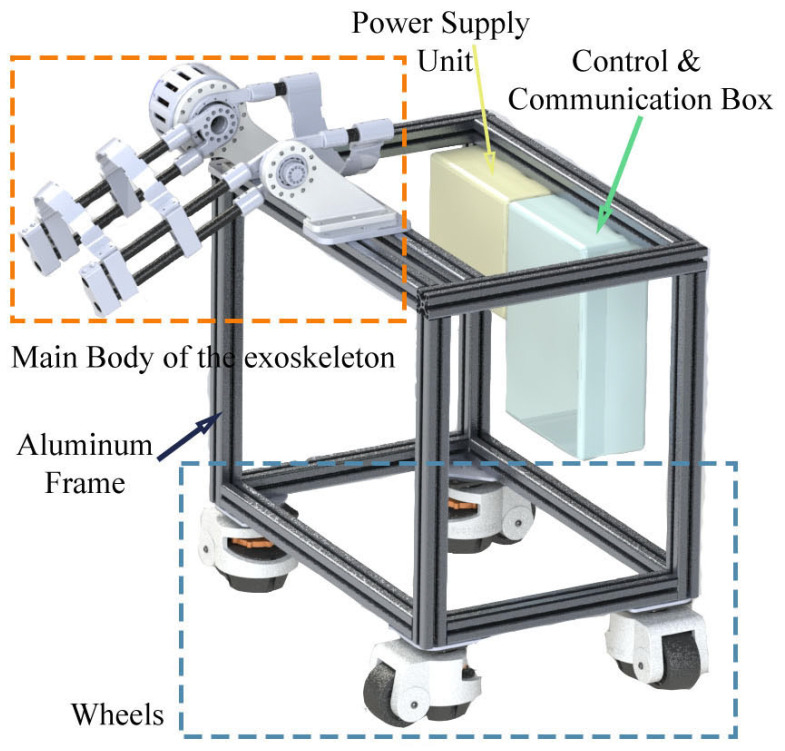
Components of the main body of the knee exoskeleton.

Figure 1 shows the structure of the exoskeleton platform. The platform comprises a sturdy aluminium frame that serves as the base structure. The frame is designed for durability and stability, enabling it to support the various components of the exoskeleton system and user. This platform can totally support 200 kg weight, which is adequate for most users. The aluminium material ensures lightweight yet rigid construction. Attached to the aluminium frame are four wheels, which allow the platform to be easily manoeuvred and positioned.

The wheels can be locked to keep the platform stationary when required. This mobility feature enhances the usability and flexibility of the exoskeleton system, allowing it to be quickly deployed and adjusted according to the user’s needs. A control and communication box is attached on the back side of the platform. In this prototype, we applied a DC 32V regulated power source for the exoskeleton and control and communication box. The control and communication box consists of three parts: main controller board (STM32F407), DC–DC voltage converter, and a CAN bus communication board.

**Figure 2:**
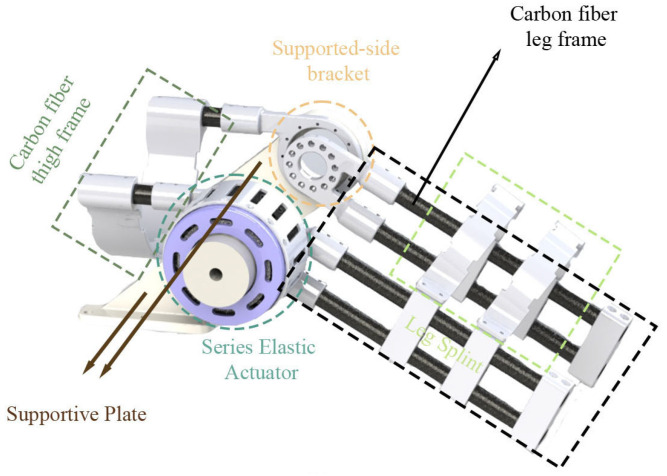
Structure of the knee exoskeleton platform.

The centrepiece of the platform is the main body of the exoskeleton, which houses the actuation system of knee joint (see Figure 2). The exoskeleton consists of a carbon fibre leg frame, which provides the base structure for the manipulator. Attached to this frame are the power supply unit and control and communication box that work together to support and assist the knee joint during rehabilitation exercises. The exoskeleton includes position-adjustable splints that can be customised to fit different users’ legs and thighs. The knee joint area is a key focus. It is designed to align with the user’s knee and provide assistance during flexion and extension movements. This mechanism includes an SEA and multiple position encoders to provide precise control and feedback.

**Figure 3:**
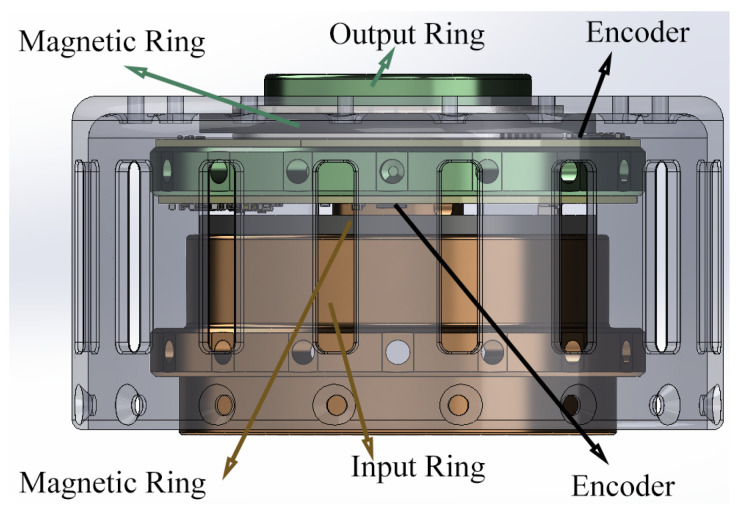
Setup of encoders in the SEA.

The exoskeleton system, equipped with the SEA, employs a cascaded proportion integration differentiation (PID) controller to precisely regulate the output position. The SEA is integrated with the exoskeleton, where the output ring of the SEA is rigidly connected to the leg frame, and the thigh frame is installed on a supportive plate fixed to an aluminium frame. To achieve accurate control and monitoring, the SEA employs two off-axis absolute encoders to measure the deflection angle θ between its input and output rings. This deflection angle is crucial for estimating the output torque of the actuator. Additionally, an on-axis absolute encoder is utilised to measure the position of the leg frame, defined as ϕ, and its angular speed, $\omega_{o}$. Figure 3 shows how the encoders are mounted in the SEA.

#### 2.1.2. Kinematic Analysis

This proposed knee exoskeleton is a single-degree-of-freedom system designed to assist knee flexion and extension movements. The primary joint, which mimics the human knee, operates as a hinge, allowing rotational motion controlled by the SEA. The kinematics of this system can be described by focusing on the rotational motion of the knee joint. Assuming a planar analysis, the motion of the leg frame relative to the thigh frame can be expressed in terms of the knee joint angle $\theta_{k}$. The position of any point on the leg frame, such as a reference point at a distance *L* from the knee joint, can be represented in the base frame as

$$p=\left[\begin{array}{c} L\cos(\theta_{k})\\ L\sin(\theta_{k}) \end{array}\right].$$

where $\theta_{k}$ varies within the physiological limits of knee motion, typically between $-120^{\circ }$ (full flexion) and $0^{\circ }$ (full extension). This range defines the workspace of the exoskeleton, which, for a fixed point on the leg frame, forms an arc of a circle centred at the knee joint. In our exoskeleton, the range of motion is designed as $-135^{\circ }$ (full flexion) in the mechanics, and a virtual boundary is set as $-120^{\circ }$ (full flexion) in the controller.

#### 2.1.3. SEA Working Principle

**Figure 4:**
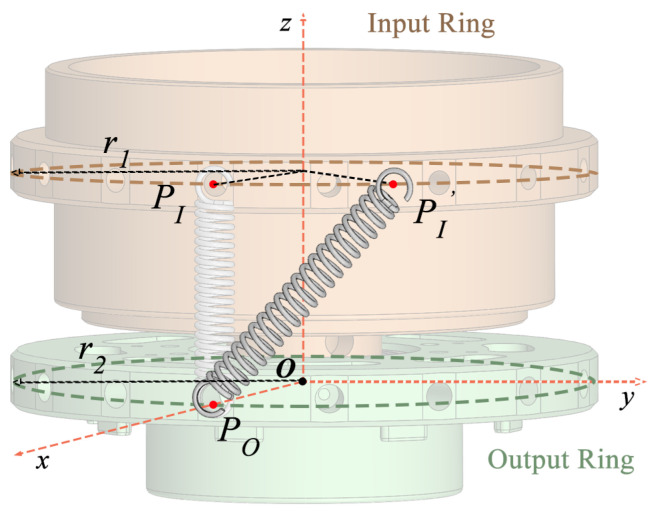
Overview of the SEA working principle.

Figure 4 depicts how the SEA functions. The input and output rings are connected through springs. Initially, at $\theta =0^{\circ }$, no torque is produced. However, once the angle $\left|\theta \right|>0$, the springs create torque that depends on their stiffness and the angle, enabling adjustments to the stiffness settings.

#### 2.1.4. Setup of the Exoskeleton

**Figure 5:**
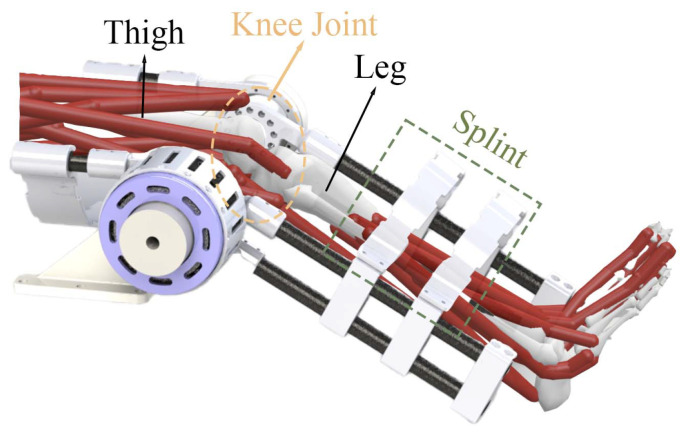
Setup of using the knee exoskeleton: user’s leg and thigh are fixed with the splints and the knee joint is aligned with the actuator output ring.

Figure 5 illustrates the integration of the exoskeleton with a human leg, highlighting the key components and their interaction. The thigh and leg splint segments of the exoskeleton are shown, with the knee joint at the centre, allowing for controlled articulation. The SEA drives the movement and is connected to the exoskeleton’s structure, providing the necessary torque for motion control. Red cables represent the artificial muscles that mimic the function of natural muscles. The leg splint, indicated by the green dashed box, stabilises the leg, ensuring proper alignment and support during rehabilitation training. This setup allows the exoskeleton to provide assistance to the participant’s knee joint, making it a tool for the rehabilitation of human motor function.

### 2.2. Exoskeleton Control

#### 2.2.1. Cascaded PID Position Controller

**Figure 6:**
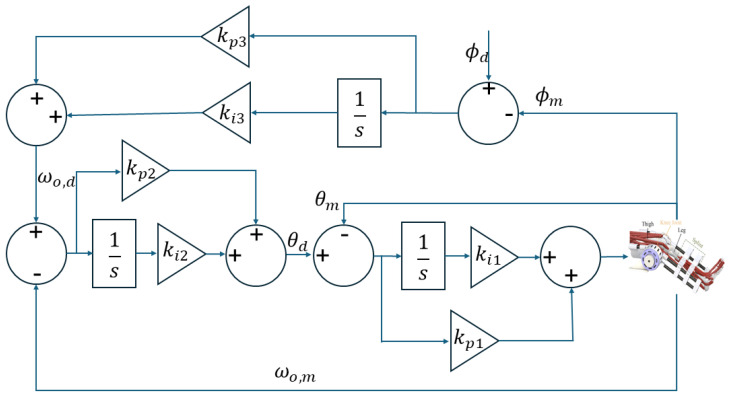
Framework of the cascaded PID controller for the position tracking control.

The cascaded PID position controller depicted in Figure 6 features a multi-loop controller designed for precise control of the output position of the actuator. The structure includes multiple loops, each providing specific control functions to ensure precise position control.

The outermost loop is directly responsible for position control. It compares the desired position $\phi_{d}$ with the measured position $\phi_{m}$ and computes the position error. This error is processed by a PI controller, which includes proportional gain $k_{p3}$ and integral gain $k_{i3}$, to generate the desired angular velocity $\omega_{o,d}$, which can be obtained as

(1) $$\omega_{o,d}=k_{p3}e_{\phi }+k_{i3}\int e_{\phi }\;dt$$

where $e_{\phi }$ is the position error and can be computed as $e_{\phi }=\phi_{d}-\phi_{m}$.

The intermediate loop works on angular velocity control of the knee joint. It takes the desired angular velocity $\omega_{o,d}$ and compares it with the measured angular velocity $\omega_{o,m}$. The resulting velocity error is fed into another PI controller with gains $k_{p2}$ and $k_{i2}$, which then produces the desired angular position $\theta_{d}$ by

(2) $$\theta_{d}=k_{p2}e_{\omega }+k_{i2}\int e_{\omega }\;dt$$

where $e_{\omega }$ is the velocity error, $e_{\omega }=\omega_{o,d}-\omega_{o,m}$.

The innermost loop is dedicated to torque control by managing the deflection angle of the SEA. It compares the desired angular position $\theta_{d}$ with the measured angular position $\theta_{m}$ and calculates the position error. This error is processed by a PI controller with gains $k_{p1}$ and $k_{i1}$ to control the deflection angle, thus regulating the output torque of the SEA.

(3) $$u=k_{p1}e_{\theta }+k_{i1}\int e_{\theta }\;dt$$

where $e_{\theta }$ is the deflection control error, which is computed as $e_{\theta }=\theta_{d}-\theta_{m}$.

#### 2.2.2. Dynamics of the Exoskeleton

The control signal *u* affects the actuator dynamics, which in turn influence the SEA’s deflection and the exoskeleton’s output position:

(4) $$J{\ddot{\theta }}_{m}+b{\dot{\theta }}_{m}+\tau_{out}=u$$

where *J* and *b* are the moment inertia and the damping coefficient of the elastic actuator.

By using this cascaded PID controller, the desired output position $\phi_{d}$ of the exoskeleton is achieved through precise control of the SEA’s deflection angle and the resulting output torque. This hierarchical control strategy ensures high precision and stability, making it suitable for complex motion control applications in exoskeletons.

#### 2.2.3. Position Control Loop

The error dynamics for the position loop are given by:

(5) $$e_{\phi }=\phi_{d}-\phi_{m}$$

The output of the PI controller is:

(6) $$\omega_{o,d}=k_{p3}e_{\phi }+k_{i3}\int e_{\phi }\;dt$$

Hence, the closed-loop transfer function for this loop can be represented as:

(7) $$G_{p}\left(s\right)=\frac{k_{p3}+\frac{k_{i3}}{s}}{s}$$

The characteristic equation of the closed-loop system is:

(8) $$s^{2}+k_{p3}s+k_{i3}=0$$

For stability, the roots of this equation must have negative real parts. By applying the Routh–Hurwitz criterion, it can be determined that the system is stable if $k_{p3}>0$ and $k_{i3}>0$.

#### 2.2.4. Velocity Control Loop

The error dynamics for the velocity loop are:

(9) $$e_{\omega }=\omega_{o,d}-\omega_{o,m}$$

The PI controller output is:

(10) $$\theta_{d}=k_{p2}e_{\omega }+k_{i2}\int e_{\omega }\;dt$$

The closed-loop transfer function for this loop is:

(11) $$G_{v}\left(s\right)=\frac{k_{p2}+\frac{k_{i2}}{s}}{s}$$

The characteristic equation of the closed-loop system is:

(12) $$s^{2}+k_{p2}s+k_{i2}=0$$

Using the Routh–Hurwitz criterion, the system is stable if $k_{p2}>0$ and $k_{i2}>0$.

#### 2.2.5. Deflection (Torque) Control Loop

The error dynamics for the torque loop are:

(13) $$e_{\theta }=\theta_{d}-\theta_{m}$$

The PI controller output is:

(14) $$u=k_{p1}e_{\theta }+k_{i1}\int e_{\theta }\;dt$$

The closed-loop transfer function for this loop is:

(15) $$G_{t}\left(s\right)=\frac{k_{p1}+\frac{k_{i1}}{s}}{s}$$

The characteristic equation of the closed-loop system is:

(16) $$s^{2}+k_{p1}s+k_{i1}=0$$

Applying the Routh–Hurwitz criterion, the system is stable if $k_{p1}>0$ and $k_{i1}>0$.

By ensuring that each individual loop is stable through appropriate tuning of the proportional and integral gains ($k_{p1},k_{i1},k_{p2},k_{i2},k_{p3},k_{i3}$), the overall cascaded PID control system will be stable. All these gains are tuned empirically. Additionally, the interactions between the loops are designed in such a way that the inner loops (which operate at higher frequencies) stabilise faster than the outer loops, thereby ensuring a stable hierarchical control structure.

#### 2.2.6. Lyapunov Stability Analysis

To further validate stability, the Lyapunov function V(e) is defined as

(17) $$V\left(e\right)=\frac{1}{2}e_{\phi }^{2}+\frac{1}{2}e_{\omega }^{2}+\frac{1}{2}e_{\theta }^{2}$$

which is positive definite and has a negative definite derivative

(18) $$\dot{V}\left(e\right)=e_{\phi }{\dot{e}}_{\phi }+e_{\omega }{\dot{e}}_{\omega }+e_{\theta }{\dot{e}}_{\theta }$$

Since each term ${\dot{e}}_{\phi },{\dot{e}}_{\omega },{\dot{e}}_{\theta }$ is negative when the gains are positive and the errors are driven to zero, $\dot{V}\left(e\right)$ will be negative definite, ensuring overall system stability.

The cascaded PID controller for the SEA in the exoskeleton is stable provided that all proportional and integral gains are positive and the inner loops stabilise faster than the outer loops, thereby ensuring a robust and precise control system for motion control applications.

### 2.3. Data Processing

#### 2.3.1. Processing of Encoder Data

The off-axis absolute encoders provide real-time measurements of the deflection angle θ, and the on-axis absolute encoder provides real-time measurements of the angular position ϕ and angular speed $\omega_{o}$ of the leg frame at the frequency of 200 Hz. Raw encoder signals may contain noise due to electrical interference, mechanical vibrations, and other disturbances. To ensure the accuracy of the control system, a first-order low-pass filter is employed to process the raw encoder data, which can be mathematically represented by

(19) y[k]=αx[k]+(1−α)y[k−1]

where y[k] is the filtered signal at the current sample *k*, x[k] is the raw signal at the current sample *k*, y[k−1] is the filtered signal at the previous sample k−1, and α is the filter constant (0<α<1), determining the degree of smoothing, which is tuned empirically.

#### 2.3.2. Calculation of Angular Speed

The on-axis absolute encoder directly measures the angular position ϕ. Differentiating the position data with respect to time provides the angular speed $\omega_{o}$. However, numerical differentiation can amplify noise, so the filtered angular position is used to calculate the angular speed of the leg frame. Additionally, Han’s Tracking Differentiator (TD) is employed to obtain a smooth estimate of the derivative (angular speed) from noisy position data [27]. The implementation involves the following steps and equations:

#### 2.3.3. Parameters

- Step size (*h*): Sampling interval.
- Tuning parameter (*r*): Controls the response speed and smoothness.

#### 2.3.4. Initialisation

Initialise the state variables:

$$\begin{array}{cc} x_{1}\left(0\right) & :\mathrm{Initial}\;\mathrm{estimate}\;\mathrm{of}\;\mathrm{the}\;\mathrm{position}.\\ x_{2}\left(0\right) & :\mathrm{Initial}\;\mathrm{estimate}\;\mathrm{of}\;\mathrm{the}\;\mathrm{derivative}\;(\mathrm{angular}\;\mathrm{speed}). \end{array}$$

#### 2.3.5. Update Equations

For each time step *k*, update the state variables using the following equations:

Error Calculation:

(20) $$e_{k}=x_{1}\left(k\right)-u\left(k\right)$$

where u(k) is the input signal (angular position) at time step *k*, the error $e_{k}$ is the difference between the current estimate of the position $x_{1}\left(k\right)$ and the actual position u(k).

Intermediate Variables:

(21) $$\begin{array}{cc} a_{0} & =h\cdot x_{2}\left(k\right) \end{array}$$

(22) $$\begin{array}{cc} y & =e_{k}+a_{0} \end{array}$$

(23) $$\begin{array}{cc} d & =r\cdot h \end{array}$$

(24) $$\begin{array}{cc} a_{1} & =\sqrt{d\cdot (d+8\cdot |y|)} \end{array}$$

(25) $$\begin{array}{cc} a_{2} & =a_{0}+\mathrm{sign}\left(y\right)\cdot \frac{a_{1}-d}{2} \end{array}$$

(26) $$\begin{array}{cc} a & =\left\{\begin{array}{cc} 0 & \mathrm{if}\;a_{0}+y=0\\ a_{2} & \mathrm{otherwise} \end{array}\right. \end{array}$$

where $a_{0}$ is a temporary variable that accounts for the influence of the current derivative estimate $x_{2}\left(k\right)$. *y* is an intermediate variable combining the error and $a_{0}$. *d* is a product of the tuning parameter *r* and the step size *h*. $a_{1}$ and $a_{2}$ are intermediate variables used to compute a non-linear adjustment *a*.

Non-linear Function:

(27) $$f_{han}=-r\cdot \frac{a}{d+|a|}$$

where $f_{han}$ is a non-linear adjustment term designed to ensure the differentiator tracks the input signal smoothly.

State Updates:

(28) $$\begin{array}{cc} x_{1}\left(k+1\right) & =x_{1}\left(k\right)+h\cdot x_{2}\left(k\right) \end{array}$$

(29) $$\begin{array}{cc} x_{2}\left(k+1\right) & =x_{2}\left(k\right)+h\cdot f_{han} \end{array}$$

The position estimate $x_{1}\left(k+1\right)$ is updated based on the current position and the estimated derivative. The derivative estimate $x_{2}\left(k+1\right)$ is updated based on the current derivative and the non-linear adjustment $f_{han}$.

The following steps are conducted to implement Han’s TD:

1. Initialise the state variables $x_{1}$ and $x_{2}$.
2. For each time step *k*:
  (a) Calculate the error $e_{k}$.
  (b) Compute the intermediate variables $a_{0}$, *y*, $a_{1}$, $a_{2}$, and *a*.
  (c) Calculate the non-linear function $f_{han}$.
  (d) Update the state variables $x_{1}$ and $x_{2}$ for the next time step.

## 3. Experiments and Results

### 3.1. Exoskeleton Comparison

The proposed exoskeleton is compared with typical knee exoskeletons in the literature as shown in Table 1.

### 3.2. Trajectory Tracking Test

**Figure 7:**
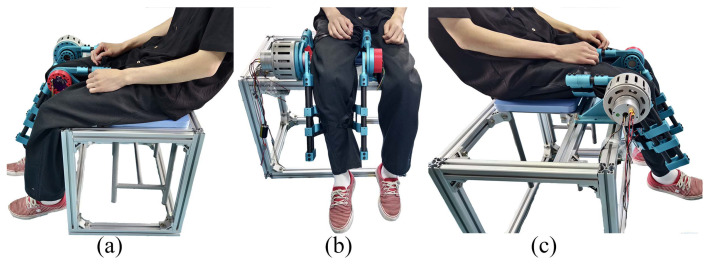
Experiment setup of the position tracking with healthy participants: (**a**–**c**) show different perspectives of the setup.

The performance of the cascaded PID controller in tracking the desired position trajectories was evaluated using two different sinusoidal signals with frequencies of 0.1 Hz and 0.05 Hz, respectively. The evaluation involved applying these trajectories to the exoskeleton system and measuring the actual output angles and the corresponding tracking errors. Figure 7 shows the setup of this test.

#### 3.2.1. 0.1 Hz Sinusoidal Trajectory

**Figure 8:**
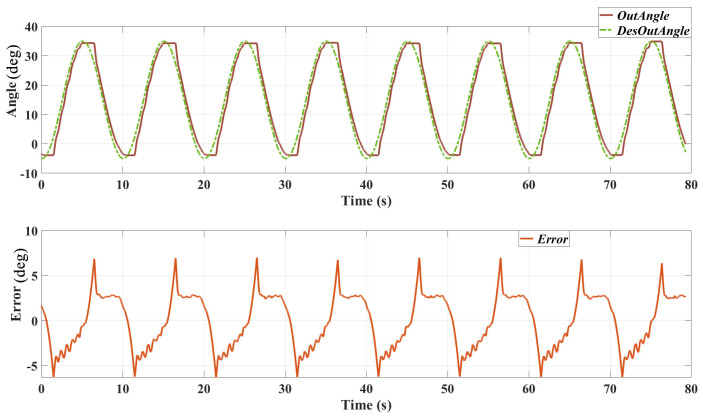
Position tracking performance with a 0.1 Hz sinusoidal trajectory. The top plot shows the desired and actual output angles, and the bottom plot shows the tracking error over time.

Figure 8 presents the results of the position tracking test with a 0.1 Hz sinusoidal trajectory. The top plot shows the desired output angle and the actual output angle over time, while the bottom plot illustrates the tracking error.

Desired vs. Actual Output Angles: the actual output angle closely follows the desired trajectory, with minor deviations observed at the peaks and troughs of the sine wave. Tracking Error: the tracking error fluctuates around zero, indicating that the controller effectively minimises the deviation from the desired trajectory. There are several reasons causing this error: 1. The bandwidth of the actuator is limited, which caused the delay in the position tracking result. 2. The friction of the mechanical system of the SEA prototype and the exoskeleton might be relatively high; thereby, the real output angle did not change when the desired position direction changed.

#### 3.2.2. 0.05 Hz Sinusoidal Trajectory

**Figure 9:**
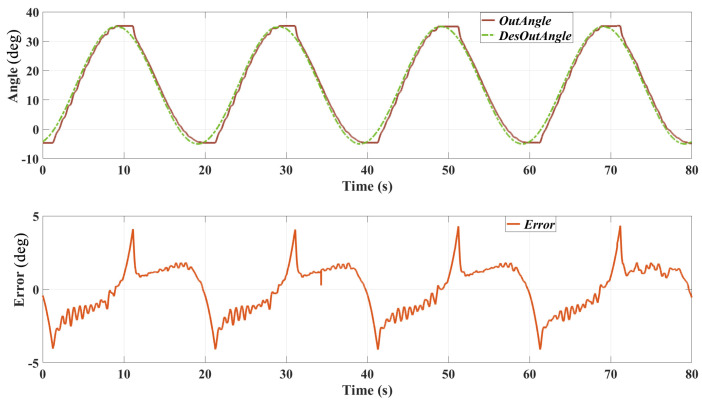
Position tracking performance with a 0.05 Hz sinusoidal trajectory. The top plot shows the desired and actual output angles, and the bottom plot shows the tracking error over time.

Figure 9 shows the results of the position tracking test with a 0.05 Hz sinusoidal trajectory. Similar to the previous test, the top plot displays the desired and actual output angles, and the bottom plot shows the tracking error.

Desired vs. Actual Output Angles: the actual output angle tracks the desired trajectory with high accuracy. The response shows smoother transitions with less noticeable deviations compared to the 0.1 Hz test. Tracking Error: the tracking error remains low throughout the test, demonstrating the controller’s ability to maintain precise position tracking at lower frequencies. The maximum error is reduced compared to the 0.1 Hz test, indicating improved performance at this frequency.

#### 3.2.3. Patients Passive Training

**Figure 10:**
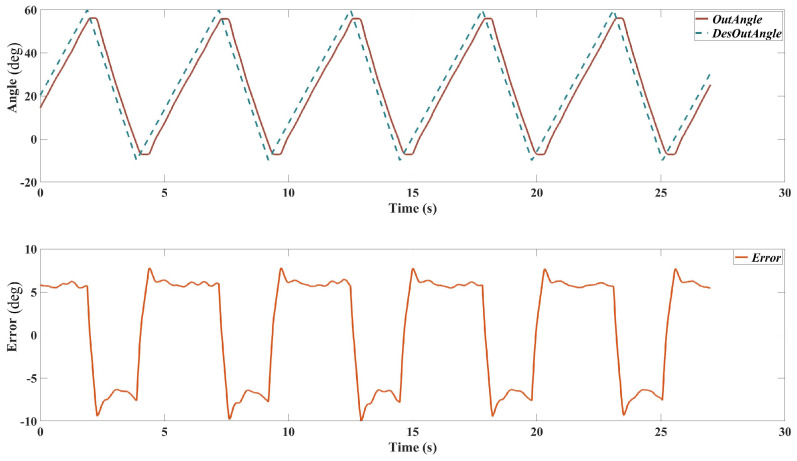
Passive training result on one stroke patient. Error=DesOutAngle−OutAngle.

To demonstrate the performance of the designed exoskeleton on real stroke patients, one patient was recruited to use the exoskeleton for passive tracking. Due to time constraints, more patients will be involved to ensure the repeatability of the result. The training trajectory is design as a triangle-shape wave to emulate the isokinetic dynamometer. The passive training test result is shown in Figure 10.

### 3.3. Clinical Test with Post-Stroke Patients

#### 3.3.1. Experiment Setup

With approval from the Ethics Committee of University of Health and Rehabilitation Sciences (KFDX:NO.2022-201), two patients with lower limb spasticity due to stroke were recruited from the Central Hospital of Qingdao. The participants were as follows: Sub0 (male, 42 years old, MAS = 2) and Sub1 (male, 60 years old, MAS = 1). The inclusion criteria for these patients were: (a) currently experiencing a stroke (either cerebral haemorrhage or cerebral infarction) and related central nervous system disorders, with spasticity in the knee extensor muscles of the lower limbs; (b) knee joint spasticity rated between 1 and 3 on MAS; (c) stable medical condition allowing for participation in the research study; (d) no history of musculoskeletal disorders affecting knee flexion and extension; (e) clear consciousness, no cognitive impairments, and the ability to understand and follow instructions of therapists.

**Figure 11:**
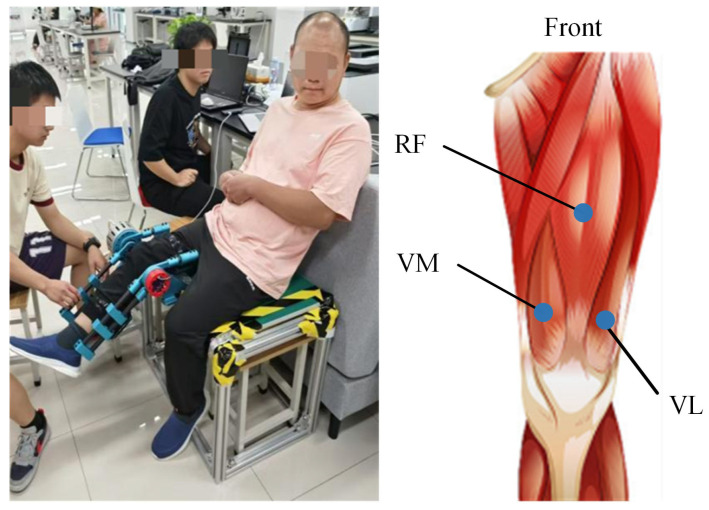
Custom-developed knee exoskeleton and the anatomical diagram of knee extensor muscles detected for quantitative analysis.

In this study, a commercial wireless biosignal acquisition system (Delsys Inc., Boston, MA, USA) was used to collect sEMG signals during knee joint rehabilitation. The sEMG signals were sampled at 2000 Hz. The sensors were positioned on the anterior thigh, encompassing three channels: VM, RF, and VL. Sensor placement followed the reference positions recommended by the SENIAM guidelines for non-invasive muscle assessment in the European Union. The sensor locations are illustrated in Figure 11. After positioning and securing the sEMG sensors, the custom-developed exoskeleton was operated to perform passive stretching of the knee joint in stroke patients to measure the muscle activity induced by the passive knee flexion. Considering that spasticity is velocity-dependent, the assessment involved passive knee flexion at two different stretch-reflex periods: slow (2s) and fast (1s). Each stretching velocity involved a 2 min stretch from knee extension to flexion. All evaluations were conducted by a physical therapist with extensive experience in assessment to minimise extraneous variability.

#### 3.3.2. Data Pre-Processing

The experiment used the exoskeleton’s proprietary angle information to define the motion cycles. The data acquisition process recorded both knee flexion and extension movements; however, a valid motion cycle should encompass only the complete knee flexion process. Consequently, during data processing, activation segment detection techniques were applied to isolate the sEMG signals related to lower limb flexion. By detecting these activation segments, the start and end times of each motion cycle was determined, allowing for the extraction of valid sEMG signals. Additionally, the sEMG signals underwent band-pass filtering to reduce noise and improve signal quality.

To analyse muscle activity during passive knee flexion, the following analyses on the preprocessed sEMG signals were conducted for each cycle: (1) calculate the root mean square (RMS) for each cycle using the specified formula; (2) compute the mean RMS for each cycle and represent it with a line graph according to the exoskeleton’s activity sequence; (3) perform a regression analysis of the RMS mean for individual cycles against the activity sequence (time) to quantify changes in sEMG signals following exoskeleton movements.

#### 3.3.3. Visualisation of RMS Mean Results

**Figure 12:**
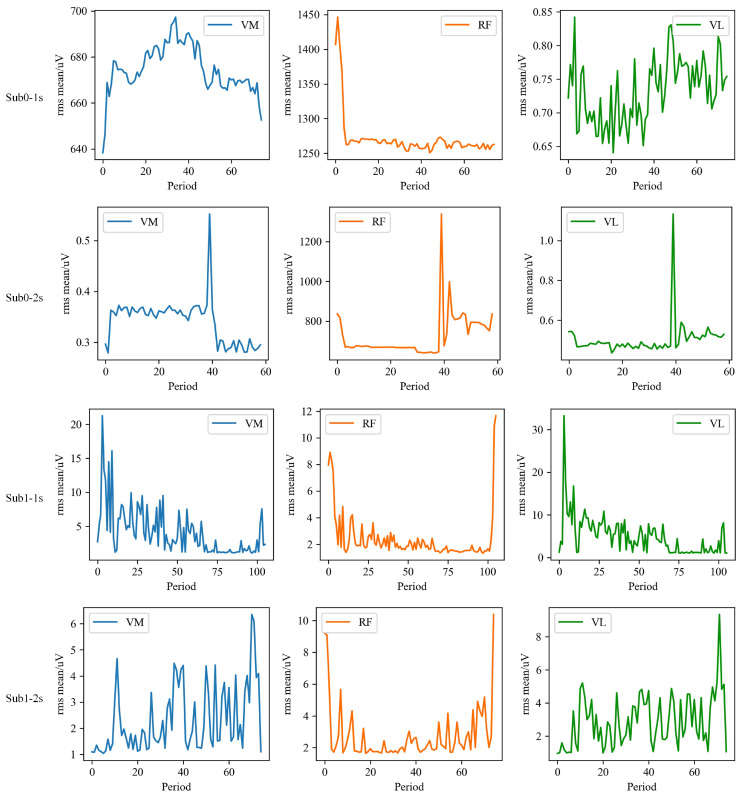
RMS mean of each muscle of Sub0 and Sub1 in two stretching velocities, i.e., fast (1 s) and slow (2 s).

To conduct a thorough analysis of sEMG signals in stroke patients when using the knee joint exoskeleton, this study focused on calculating the RMS mean for each cycle. The RMS mean across different channels was then visualised using line graphs, as illustrated in Figure 12. This method aims to clearly depict the trends in sEMG signal variations across different channels and their interrelationships, providing valuable data for further analysis and interpretation. Specifically, in Sub0, the muscle tone in the initial segment of the RF during fast and slow stretching modes shows a decreasing trend. Furthermore, in Sub1, this reduction in muscle tone is more pronounced in the VM, RF, and VL during fast stretching, suggesting that the exoskeleton intervention effectively alleviated muscle tone.

#### 3.3.4. Exoskeleton’s Impact on Muscle Tone

**Figure 13:**
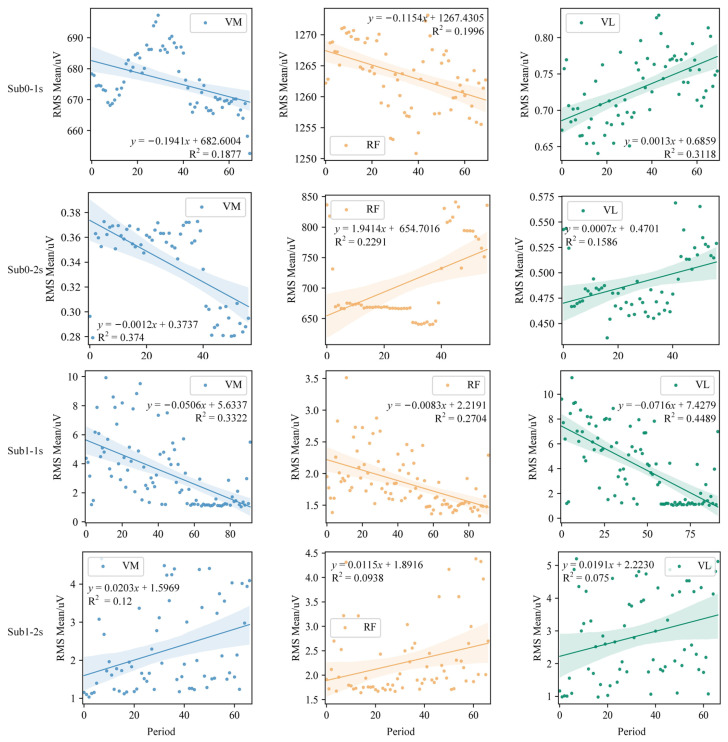
Regression results between the RMS mean of each muscle and the segment number in two stroke patients.

To assess the impact of the exoskeleton on muscle tone in stroke patients, a regression analysis of the RMS mean was conducted for individual cycles against the number (time) of activity segments, with results depicted in Figure 13. The analysis reveals that in the Sub0 group, the RMS mean of VM and RF in the fast stretching mode, as well as VM in the slow mode, showed a negative correlation during training, supporting the exoskeleton’s efficacy in reducing muscle tone. The regression analysis indicates that each exoskeleton activity reduced muscle tone in stroke patients by between −0.1941 and −0.0012 μV. By contrast, the regression analysis for VL in the two stretching modes revealed a positive correlation, though with smaller slopes of 0.0013 and 0.0007, respectively. Notably, the RMS mean values for VL are significantly lower than those for VM and RF, especially in the fast stretching mode. As for the Sub1 group, VM, RF, and VL in the fast stretching mode all exhibited a negative correlation. Further analysis showed that each exoskeleton activity reduced muscle tone by −0.0716 to −0.0083 μV, although at 2 s, a positive correlation was observed.

#### 3.3.5. Impact of Passive Activity Velocity

**Figure 14:**
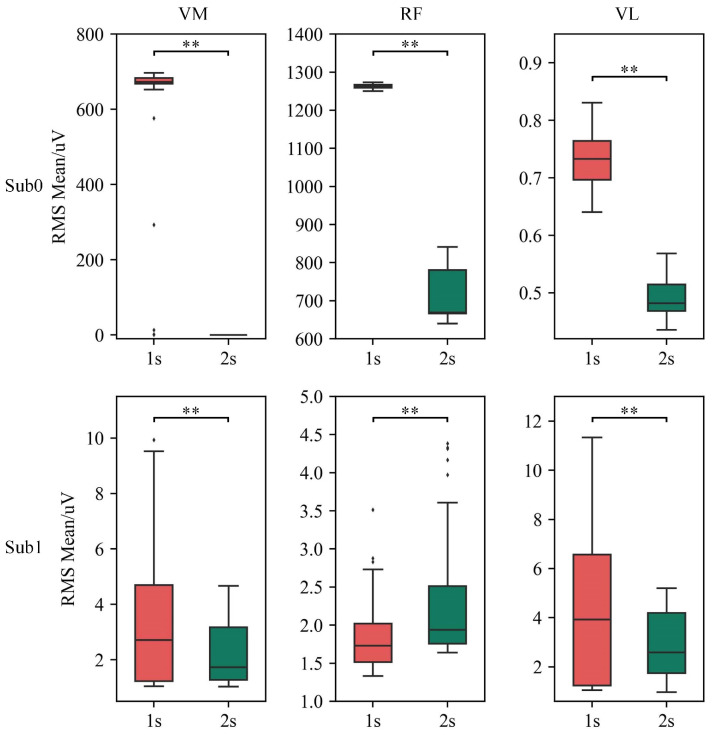
Comparison of the RMS mean of each muscle at different passive activity velocities. ** denotes *p* < 0.01.

Analysis of RMS mean values at different passive activity velocities is presented in Figure 14. The results demonstrate that at a faster passive activity velocity (1 s), the RMS mean values are significantly higher in VM, RF, and VL for the Sub0 group, as well as in VM and VL for the Sub1 group, compared to those at a slower passive activity velocity (2 s). This difference is statistically significant (*p* < 0.01).

## 4. Discussion

### 4.1. Analysis on Trajectory Tracking

The results from the position tracking tests demonstrate the effectiveness of the cascaded PID controller in accurately following desired trajectories. The controller performs well at both 0.1 Hz and 0.05 Hz frequencies, with the actual output angles closely matching the desired trajectories and maintaining relatively low tracking errors. However, the tracking error ranges from about −7 to $7^{\circ }$, which is higher than that of 0.1 Hz (which is about −4 to $4^{\circ }$), especially at the peaks and troughs of the sine wave where the trajectory changes direction rapidly. This may be caused by the friction of the system and probably indicates a limitation in the controller’s responsiveness.

The relatively high tracking error, particularly at higher frequencies, can be attributed to several factors. One significant factor is the non-linearity of the SEAs. SEAs introduce non-linear dynamics due to their elastic components, which can cause delays and oscillations in the control response. The linear nature of the PID controller may not fully compensate for these non-linear effects, leading to increased tracking errors. Additionally, the physical properties of the exoskeleton system, including inertia and damping, affect the responsiveness of the control system. Rapid changes in the desired trajectory can lead to overshoot and oscillations if the system cannot respond quickly enough.

To improve the performance of the position tracking control, especially in the presence of non-linear dynamics, several approaches can be considered. Implementing non-linear controllers, such as Sliding Mode Control (SMC) or Model Predictive Control (MPC), can better handle the non-linearities of SEAs. These controllers can adapt to changing system dynamics and provide more precise control. Furthermore, incorporating a feedforward control component can improve the system’s response to known trajectory changes. By predicting the necessary control actions ahead of time, the feedforward controller can reduce the lag and overshoot associated with rapid trajectory changes. In addition, adaptive control techniques can also be beneficial. By adjusting the controller parameters in real-time based on the system’s behaviour, adaptive control can help maintain optimal performance despite changes in system dynamics or external disturbances. Additionally, applying advanced filtering techniques to the feedback signals can reduce the impact of noise and improve the accuracy of the position measurements, helping the controller maintain more precise control.

### 4.2. Analysis on Clinical Performances

Experimental results showed that the developed exoskeleton significantly reduced muscle tone in stroke patients after multiple passive activities. Specifically, at faster passive activity velocities, the exoskeleton demonstrated a marked reduction in antagonist muscle tone. Detailed analysis of individual cycles revealed two main aspects of muscle tone reduction: first, a decrease in the RMS mean value of antagonist muscle EMG signals, indicating a notable reduction in muscle tone intensity during activity; and second, a reduction in activation time, reflecting a significant shortening of muscle activation duration. Further analysis reveals that for individual subjects, the RMS mean increases with the velocity of passive activity. This trend is observed in VM, RF, and VL for the Sub0 group, and in VM and VL for the Sub1 group, which is consistent with the velocity-dependent nature of post-stroke spasticity. Notably, in the Sub1 group, the relative contributions of different spasticity channels shift with changes in velocity. For example, at a passive activity velocity of 2 s, the primary order of muscle spasticity is VL, RF, and VM; whereas at 1 s, the order shifts to VL, VM, and RF. This suggests that varying the exoskeleton’s velocity parameters could trigger different patterns of muscle spasticity, potentially providing valuable insights for clinical treatment and evaluation.

Detailed analysis of spasticity channels reveals significant differences in the primary muscles responsible for spasticity among stroke patients. For instance, in the Sub0 group, the primary channel for knee joint spasticity is CH2, while in the Sub1 group, the main channels are CH1 and CH3. These differences suggest that various spasticity channels may be closely linked to knee joint dysfunction. Identifying these primary spasticity channels offers valuable insights for clinical evaluation, helping to pinpoint the key muscle groups involved in knee joint issues. This discovery not only aids in optimising pharmacological treatments, but also informs the development of more effective exercise training programs, allowing for a personalised approach to manage spasticity and improve overall treatment outcomes. However, several factors should be further focused in future work. Firstly, the exoskeleton can only provide training for patients with right-side impairments, preventing the verification of whether the dominant and non-dominant sides exhibit different outcomes under identical experimental conditions. Specifically, it remains unclear whether antagonistic muscles display increased tension when patients perform faster passive movements under the exoskeleton’s traction and whether this tension decreases with increased training time at the same velocity. Additionally, due to time constraints and the limited number of patients, the generalisability of the results to a broader patient population could not be tested.

## 5. Limitations

The proposed single-degree-of-freedom exoskeleton does not exhibit the typical singularities seen in higher-degree-of-freedom robotic systems. However, one notable limitation of the current design is its inherent dependency on the alignment between the exoskeleton’s joint and the user’s anatomical knee joint. Misalignment, even by a small degree, can introduce discomfort, reduce motion efficiency, and increase the risk of joint stress or injury during prolonged use. Additionally, the reliance on a single-degree-of-freedom hinge mechanism restricts the exoskeleton’s capability to accommodate natural knee joint translations, which occur due to the rolling and sliding motions of the femur over the tibia during flexion and extension. This simplification, while mechanically straightforward, may lead to deviations from the natural kinematics of the knee, especially in more dynamic activities, such as running or climbing stairs. Addressing these issues would require the incorporation of additional degrees of freedom or more sophisticated mechanisms that replicate complex knee joint behaviours. These factors must be considered in the design and control of the exoskeleton to ensure smooth and reliable operation.

## 6. Conclusions and Future Work

In this study, a novel knee exoskeleton was developed to provide high-frequency treatments and complete more treatment tasks within a shorter time frame, aiming to reduce the negative effects of therapist fatigue on treatment outcomes. The exoskeleton consists of three main parts: a leg splint, a thigh splint, and an actuator. The range of motion of this exoskeleton is $0^{\circ }$ to $135^{\circ }$. The proposed SEA is applied to power the exoskeleton and provide compliant physical human–robot interaction. A cascaded PI controller is designed to achieve the position tracking control of the exoskeleton, and tests are conducted to evaluate the performance of the controller, including the robot with and without human participants. In addition, the positive findings on the exoskeleton’s clinical benefits underscore its potential as an effective therapeutic device for addressing knee joint spasticity in stroke patients.

Although the current cascaded PID controller demonstrates good performance in position tracking, the presence of non-linear dynamics in the SEA and limitations of the PID controller may lead to relatively high tracking errors, especially at higher frequencies. To address these challenges, incorporating non-linear control strategies, adaptive control, feedforward control, enhanced feedback filtering, and system identification and compensation techniques can significantly improve the tracking performance and robustness of the exoskeleton system. Further research and experimentation with these advanced control methods will help achieve more precise and reliable position control. Moreover, to bolster the preliminary results in clinics and establish the broader applicability of the exoskeleton, we plan to expand the sample size and include a more diverse group of participants to further validate the performance and therapeutic benefits of the exoskeleton. In addition, we will also include additional evaluation metrics, such as patient satisfaction and rehabilitation efficiency, and incorporate these measures to provide a more holistic evaluation of clinical impact on patients’ rehabilitation outcomes.

## Figures and Tables

**Table 1:** Comparison between the proposed exoskeleton and the typical knee exoskeletons in the literature.

Type	DoFs	Actuation	Portable	Max Knee Cont. Torque (Nm)	Assist Early Stage Patient	On-Bed Training
This study	1	Compliant	No	42	Yes	Yes
ABLE [28]	1	Rigid	Yes	N/A	No	No
Indego [29]	2	Rigid	Yes	N/A	No	No
P.REX [30]	1	Rigid	Yes	15.7	No	No
ATLAS [31]	1	Compliant	Yes	30	No	No
EICOSI [32]	1	Rigid	Yes	18	No	No
ABLE-KS [8]	1	Rigid	Yes	30	No	No

## Data Availability

The raw/processed data required to reproduce these findings cannot be shared at this time as the data also forms part of an ongoing study.

## List of Variables

**Variable**	**Description**
θ	Deflection angle between SEA input and output rings
ϕ	Position of the leg frame
$\omega_{o}$	Angular speed of the leg frame
$\theta_{k}$	Knee joint angle
*L*	Distance from the knee joint to a reference point
$\phi_{d}$	Desired position of the leg frame
$\phi_{m}$	Measured position of the leg frame
$\omega_{o,d}$	Desired angular velocity of the leg frame
$\omega_{o,m}$	Measured angular velocity of the leg frame
$\theta_{d}$	Desired angular position for deflection angle
$\theta_{m}$	Measured angular position for deflection angle
$e_{\phi }$	Position error, $e_{\phi }=\phi_{d}-\phi_{m}$
$e_{\omega }$	Velocity error, $e_{\omega }=\omega_{o,d}-\omega_{o,m}$
$e_{\theta }$	Deflection control error, $e_{\theta }=\theta_{d}-\theta_{m}$
$k_{p1},k_{i1}$	Gains for the deflection (torque) control loop
$k_{p2},k_{i2}$	Gains for the velocity control loop
$k_{p3},k_{i3}$	Gains for the position control loop
*J*	Moment of inertia of the elastic actuator
*b*	Damping coefficient of the elastic actuator
$\tau_{out}$	Output torque of the SEA
*u*	Control signal
